# MULTIMORBIDITY COMBINATIONS AND DEPRESSIVE SYMPTOM TRAJECTORIES AMONG US OLDER ADULTS

**DOI:** 10.1093/geroni/igae098.1161

**Published:** 2024-12-31

**Authors:** Nicholas Bishop, Kealie Walker, Corey L Nagel, Jason Newsom, Anda Botoseneanu, Heather Allore, Ana Quiñones

**Affiliations:** University of Arizona, Tucson, Arizona, United States; University of Arizona, Tucson, Arizona, United States; University of Arkansas for Medical Sciences, Little Rock, Arkansas, United States; Portland State University, Portland, Oregon, United States; University of Michigan-Dearborn, Dexter, Michigan, United States; Yale University, New Haven, Connecticut, United States; Oregon Health & Science University, Portland, Oregon, United States

## Abstract

Multimorbidity (2+ chronic health conditions) is directly related to increased patient burden, but the association between specific multimorbidity combinations and trajectories of depressive symptoms among older adults is unknown. We used data from the Health and Retirement Study (HRS) to estimate the association between multimorbidity combinations identified in 2012 and trajectories of depressive symptoms from 2012-2020 in adults aged ≥ 65 (n=9,004; mean age: 75.3; % female: 58.7%). Self-reported physician-diagnosed conditions included arthritis, hypertension, diabetes, heart conditions, cancer, lung disease, and stroke. The eight-item CES-D measured depressive symptoms. Multimorbidity combinations were used to predict quadratic latent growth trajectories of depressive symptoms, adjusting for sociodemographic, behavioral, and childhood characteristics and the complex sampling design of the HRS. In 2012, 75.6% of respondents experienced multimorbidity and the average depression score was 1.4 (SD=1.9). Nine unique multimorbidity patterns were identified, most common being hypertension+arthritis (14.6%), hypertension+heart conditions+arthritis (7.1%), and hypertension+diabetes+arthritis (6.1%). Several multimorbidity combinations were associated with greater baseline depressive symptomatology (e.g., compared to those reporting no conditions at baseline, those reporting hypertension+lung disease+arthritis had 0.96 more baseline depressive symptoms (SE=0.2, p<.001), and those reporting hypertension+diabetes+heart conditions+arthritis had 0.93 more baseline depressive symptoms (SE=.2, p<.001), respectively). Several multimorbidity combinations were associated with linear and nonlinear change in depressive symptoms, with hypertension+cancer+arthritis and hypertension+diabetes+arthritis being associated with the most rapid increases in depressive symptoms. Our work highlights the increased risk of depression among older adults with varied multimorbidity combinations and speaks to the mental health burden associated with multimorbidity.

